# Levels of Lycopene β-Cyclase 1 Modulate Carotenoid Gene Expression and Accumulation in *Daucus carota*

**DOI:** 10.1371/journal.pone.0058144

**Published:** 2013-03-29

**Authors:** Juan Camilo Moreno, Lorena Pizarro, Paulina Fuentes, Michael Handford, Victor Cifuentes, Claudia Stange

**Affiliations:** 1 Departamento de Biología, Facultad de Ciencias, Universidad de Chile, Santiago, Chile; 2 Max Planck Institut für Molekulare Pflanzenphysiologie, Potsdam-Golm, Germany; 3 Departamento de Ecología, Facultad de Ciencias, Universidad de Chile, Santiago, Chile; Kyushu Institute of Technology, Japan

## Abstract

Plant carotenoids are synthesized and accumulated in plastids through a highly regulated pathway. Lycopene β-cyclase (LCYB) is a key enzyme involved directly in the synthesis of α-carotene and β-carotene through the cyclization of lycopene. Carotenoids are produced in both carrot (*Daucus carota*) leaves and reserve roots, and high amounts of α-carotene and β-carotene accumulate in the latter. In some plant models, the presence of different isoforms of carotenogenic genes is associated with an organ-specific function. *D. carota* harbors two *Lcyb* genes, of which *DcLcyb1* is expressed in leaves and storage roots during carrot development, correlating with an increase in carotenoid levels. In this work, we show that DcLCYB1 is localized in the plastid and that it is a functional enzyme, as demonstrated by heterologous complementation in *Escherichia coli* and over expression and post transcriptional gene silencing in carrot. Transgenic plants with higher or reduced levels of *DcLcyb1* had incremented or reduced levels of chlorophyll, total carotenoids and β-carotene in leaves and in the storage roots, respectively. In addition, changes in the expression of *DcLcyb1* are accompanied by a modulation in the expression of key endogenous carotenogenic genes. Our results indicate that *DcLcyb1* does not possess an organ specific function and modulate carotenoid gene expression and accumulation in carrot leaves and storage roots.

## Introduction

**Figure 1:**
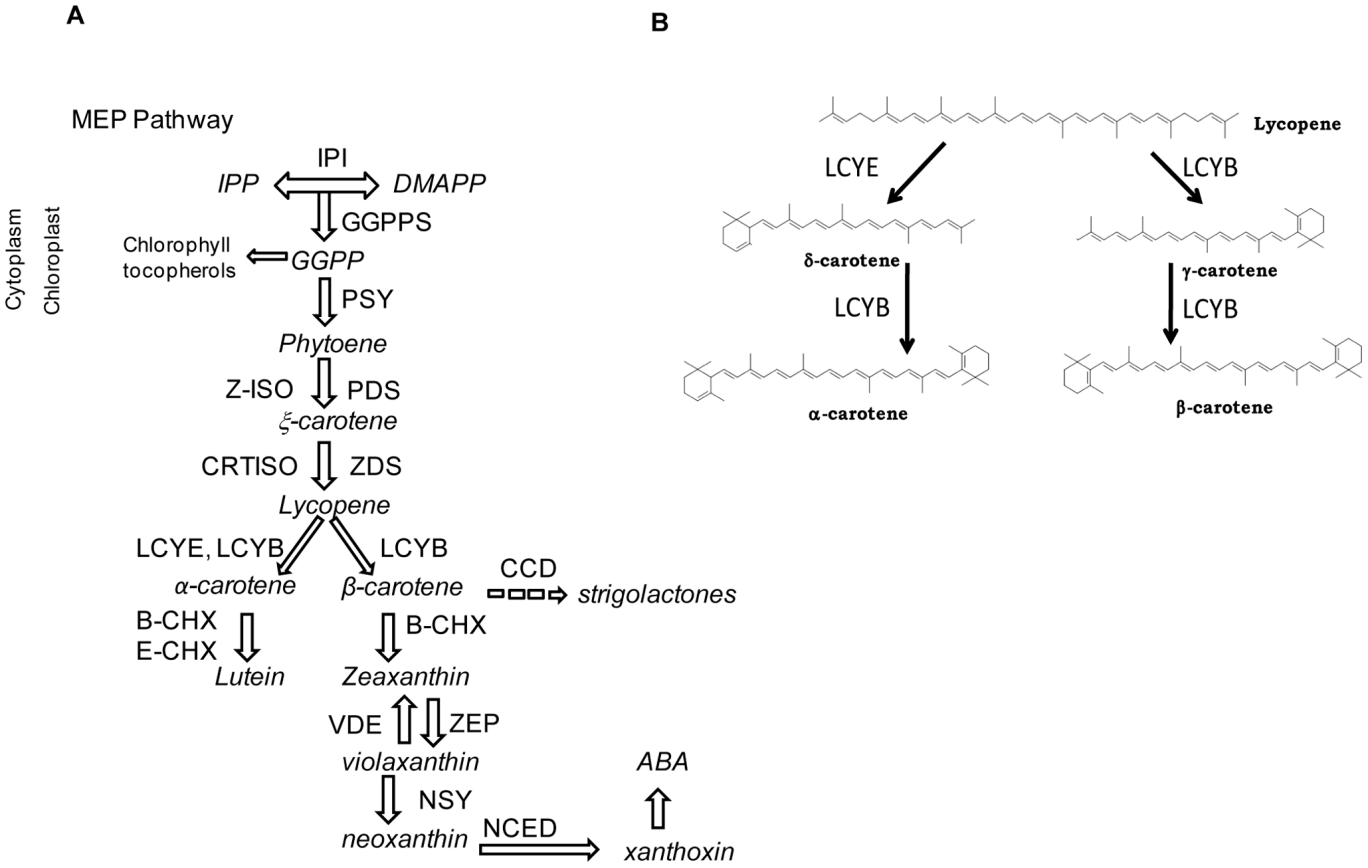
Schematic diagram of carotenoid synthesis in plants. (A) The carotenoid biosynthetic pathway in plants. Enzymatic conversions are shown by arrows with the enzymes involved in each reaction. GGPPS: Geranyl-Geranyl Pyrophosphate Synthase; PSY: Phytoene Synthase; PDS: Phytoene Desaturase; ζ-ISO: ζ-carotene Isomerase; ZDS: ζ-carotene Desaturase; CRTISO: Carotenoid Isomerase; Z-ISO: ζ-carotene isomerase, LCYB: Lycopene β-cyclase; LCYE: Lycopene ε-cyclase; B-CHX: β-carotene Hydroxylases; E-CHX: ε-carotene Hydroxylases; ZEP: ZeaxanthinEpoxidase; VDE: Violaxanthin de-epoxidase; NSY: Neoxanthin synthase, CCD: Carotenoid cleavage dioxygenase; NCED: 9-cis-epoxycarotenoid dioxygenase. (B) Lycopene β-cyclase Reaction. The LCYB enzyme transforms one molecule of lycopene into one molecule of β-carotene or α-carotene (together with LCYE) using NADPH as cofactor.

Carotenoids are isoprenoid pigments synthesized in plants, algae and some bacteria and yeast. In chloroplasts of photosynthetic organs, they play classical roles in several processes such as light absorption during photosynthesis, photo-protection via energy dissipation and reactive oxygen species (ROS) detoxification. Carotenoids also provide yellow, orange and red colors to fruits and flowers for animal-mediated pollination and seed dispersal. In these organs, carotenoids are synthesized and stored in specialized plastids called chromoplasts. In addition, carotenoids act as precursors of important apocarotenoids such as the growth regulators abscisic acid (ABA, Figure 1A) and strigolactones [1], [2], [3], [4] and of volatile flavour/aroma terpenes [5].

In animals they are fundamental for health and must be taken in their diets as an essential source of retinoids and vitamin A [6], [7]. In humans, carotenoids have been shown to have antioxidant-promoting activities [8], [9].

Carotenoids are derived from secondary metabolic processes that take place in plastids, and even though almost all of the nuclear encoded carotenogenic genes have been identified [10], [11], there is still limited knowledge concerning the regulation of the carotenoid pathway. Carotenoids are synthesized using IPP derived from the non-mevalonate pathway (MEP) as substrate to produce the common C20 precursor, geranylgeranyl diphosphate (GGPP). Two molecules of GGPP are substrates of phytoene synthase (PSY), the first committed step in carotenoid biosynthesis (Figure 1A). The colorless phytoene is then modified to form the reddish lycopene through sequential desaturations and isomerizations carried out by the enzymes phytoene desaturase (PDS), ζ–carotene desaturase (ZDS), carotenoid isomerase (CRTISO) and ζ–carotene isomerase (ζ-ISO)[12], [13]. Hydroxylation of β-carotene and α-carotene results in the synthesis of xanthophylls such as violaxanthin, zeaxanthin and lutein (Figure 1A). Lycopene cyclization is accomplished by lycopene β-cyclase (LCYB), which produces β-carotene (two β-ionone rings) in the presence of NADPH as co-factor, whereas α-carotene is created by LCYB and lycopene ε-cyclase (LCYE)(Figure 1B) [14], [15], [16]. The partial pyridine nucleotide binding site is universally present in the lycopene cyclase enzymes, despite the evolutionary divergence between lycopene cyclases of non-photosynthetic bacteria and those of photosynthetic organisms [17], [18]. In plants, unlike in bacteria and cyanobacteria, carotenoid enzymes have a signal peptide in the amino terminus for import into the plastids [8], [19], [20], [21], [22]. Specifically, the signal peptide in lycopene β-cyclase is found in the first 50–100 amino acids [21], [22]. In LCYBs of plants and bacteria, a conserved “dinucleotide binding motif” is also found. In addition, several conserved domains, such as the “Conserved region β- LCY's”, Cyclase Motif I and II, and a charged region are highly conserved in plants and cyanobacterial LCYBs, and partially conserved in bacterial LCYBs, whereas a domain named “β-cyclase motif” is fully conserved in all plants LCYBs. These motifs and regions could be involved in the substrate-enzyme interaction, in membrane association and in catalysis [17], [22], [23].

In several plant models such as *Arabidopsis thaliana* [24], *Oryza sativa* [25] http://rice.plantbiology.msu.edu/),and *Zea mays* [26], [27], LCYB is encoded by a single gene. Nevertheless, two genes encoding lycopene β-cyclase have been found in some plants that accumulate high levels of carotenoids in non-photosynthetic organs, such as fruits and flowers. These genes are differentially expressed in photosynthetic and non-photosynthetic organs. In tomato, *SlLcyb* is preferentially expressed in green organs, while *SlCycb* in ripening fruits and flowers [28], [29]. Similar organ specific functions of two *Lcyb* genes have also been described in *Capsicum annuum* (pepper; [22]), *Carica papaya* [30], [31], [32], *Croccus sativus* [33] and *Citrullus lanatus* [16].

In order to determine the function of carotenoid genes, several mutants have been described such as *Lcyb* deleterious hemizygous mutants in *Oryza sativa*, named “pre-harvested stress phenotype” (*phs4-1* and *phs4-*2) and the insertional mutant PS1 in *Zea mays* [25], [27]. These mutants show a phenotype that resembles that of others which are defective in carotenoid synthesis, such as *Psy*, *Pds* and *Zds* mutants, which display leaf bleaching, ROS accumulation and viviparity due to a deficiency in ABA synthesis [25], [27], [34], [35], [36], [37], [38].

In carrots (*Daucus carota*), β-carotene and α-carotene accumulate to high levels in the storage root, a non-photosynthetic organ [8], [39], [40], and in the leaves [8], [39], [41]. Just et al., 2007 [42] identified complete cDNA sequences for most of the carotenogenic genes of *D. carota*, of which two are candidate sequences to encode for lycopene β-cyclase, *DcLcyb1* (DQ192190) and *DcLcyb2* (DQ192191). *DcLcyb1* is transcriptionally regulated; expression studies showed that *LCYB1* transcription increases significantly during storage root development in coordination with an increase in carotenoid and β-carotene levels [39], [41]. This expression is affected differentially by light treatments in distinct developmental stages of the root [41].

Here, we compared *DcLcyb1* expression in leaves and roots, showing that the expression level is higher in the aerial organs throughout *D. carota* development. In addition, we analyzed *DcLcyb1* function by means of heterologous complementation in *E. coli*, subcellular localization, post-transcriptional gene silencing and over-expression in *D. carota*. We conclude that this gene encodes for a functional plastid-targeted LCYB and is required for β-carotene synthesis in both leaves and storage roots of *D. carota*. Moreover, *DcLcyb1* participates in the regulation of endogenous *DcPsy1, DcPsy2* and *DcLcyb2* carotenogenic genes.

## Methodology

### Plant Material

Seeds of commercially-acquired carrot *(Daucus carota L.)* cultivar Nantaise were sown in a mix of soil and vermiculite (2∶1) and cultivated in a greenhouse for 4, 8 and 12 weeks with a 16 h long day photoperiod illuminated with cool-white fluorescent light (115 μmol m^−2^ s^−1^) at 20–23°C until molecular analysis were carried out. Hypocotyls of four-week-old *in vitro* wild-type carrot plantlets cultivated in solidified MS (Murashige and Skoog) medium were harvested and utilized in *Agrobacterium*-mediated transformation experiments. Transformed carrots were transferred to pots (20×10) and cultivated in the greenhouse, as described above, when they reached 5 cm long.

### RNA extraction and quantitative RT-PCR

Total RNA was extracted from a frozen powder of 200 mg of *D.carota* leaves and storage roots of 4, 8 and 12 week-old plants using RNA solv (Omega Biotec, USA). For cDNA synthesis, 2 *μ*g of total DNA-free RNA was mixed with1mM of oligodT primer and Impron II reverse transcriptase (Promega).

Quantitative RT-PCR (qRT) experiments were performed as described in [43] in a Stratagene Mx3000P thermocycler, using SYBR Green double strand DNA binding dye. Specific primers were designed targeting the 5′ UTR of *DcLcyb*1 (Access N° DQ192190), *DcPsy1* (Access N° DQ192186), *DcPsy2* (Access N° DQ192187), *DcLcyb2* (Access N° DQ192191) and for the coding sequence of the carrot *ubiquitin* gene (Access N° DCU68751), selected as the normalizer are listed in Table 1. Final data were obtained introducing fluorescence results in the equation described by [44]. Each qRT-PCR reaction was performed with three biological replicates and each sample was analyzed in duplicate (technical replicate). In all cases, the reaction specificities were tested with melting gradient dissociation curves and electrophoresis gels. To test for significant differences in gene expression, results were analyzed using the General Linear Models option in the statistical software package Graphpad Prism. The one and two tailed Student t-test (p<0.05, confidence interval 95%), were used.

**Table 1:** Gene-specific primers used for functional characterization of *DcLcyb1*.

Gene	Primer name	Sequence (5′–3′)	Purpose
*Lcyb*	*Lcyb*1Fq *Lcyb*1Rq	tgagtgcagcttacacctacttgatta aactgcagaagatattggaga	To measure the expression of *DcLcyb1* gene by qRT
*Psy1*	*Psy1*Fq *Psy1*Rq	agtcgatggagcattaccataattc ctaatgggttacagagggttgtgtta	To measure the expression of *DcPsy1*gene by qRT
*Psy2*	*Psy2*Fq *Psy2*Rq	gctaataaacttccgtgggtgttc gctggagttagtgctaccc	To measure the expressionof *DcPsy2*gene by qRT
*Lcyb2*	*Lcyb2*Fq *Lcyb2*Rq	gattcctctgtgtccatatctcccgattgcccagaaagactcaacag	To measure the expression of *DcLcyb2* gene by qRT
*Ubi*	ubiF ubiR	gctcgaggacggcagaac cttgggcttggtgtaggtcttc	Normalizer gene for qRT experiments
*Lcyb*	*Lcyb*Fatg *Lcyb*R	atgaaagtgatggatactctac cttcacaagcattttgaactag	To amplify the complete cDNA of *DcLcyb1* for over expression and complementation
*Lcyb*	*LcybF*S *LcybR*S	gaattctatggtgtttgggtggatgga gaattcaggaatgtagggattttaactg	To amplify a coding *DcLcyb1* fragment for silencing
*HptII*	*HptII*F *HptII*R	tttgtgtacgcccgacagt aagacctgcctgaaaccga	To determine transgenic lines
*Lcyb*	FLcybi RLcybi	gggttagcggtagcacaac gcctctgcctgtactccctc	To determine*DcLcyb1* silenced lines
*Lcyb*	*Lcyb*F *Lcyb*R	Atgaaagtgatggatactctac cttcacaagcattttgaac	To amplify *DcLcyb1* gene for sub-cellular localization

### Heterologous complementation in *Escherichia coli*

The *DcLcyb1* cDNA from *D. carota* was obtained by means of conventional RT-PCR in the presence of Impron II reverse transcriptase (Promega®) using RNA extracted from leaf and root. The primers used for PCR amplification of *DcLcyb1* (1520 bp) were *Lcyb*Fatg and *Lcyb*R (Table 1). The amplified fragment was cloned into pCR®8/GW/TOPO (Invitrogen) following the manufacturer's instructions. Positive clones obtained by enzymatic digestion were sequenced in Macrogen Corp. (USA). From the pCR8/*Lcyb1* construct, *DcLcyb1* was amplified using *Lcyb*Fatg and *LCYBR* in the presence of Pfu (Fermentas) and cloned in the *EcoR*V site of the pET-Blue1 (NovaBlue®) expression vector. Positive clones that harbor the gene in the sense orientation with respect to the T7 promoter were selected by enzymatic digestion, creating pET-Blue1/*Lcyb1*.

Functional assays were carried out in *Escherichia coli* BL 21 gold strain transformed with the pDS1B or pDS1BΔ*crtY* plasmids. pDS1B carries the carotenogenic genes of *Erwinia uredovora* required to produce β-carotene [45]. The pDS1BΔ*crtY* vector has a mutation in the *crtY (Lcyb)* gene, which leads to the accumulation of lycopene in this strain [45].

This mutant strain was transformed with pET-Blue1/*Lcyb1* and the empty, pET-Blue1 vector as a control. The transformed colonies were selected in LB medium supplemented with ampicillin (100 µg mL^−1^) and chloramphenicol (50 µg mL^−1^), after an incubation of 96 hours at 30°C. An overnight liquid culture of the mutant strain transformed with pET-Blue1/*DcLcyb1*or pET-Blue1 plasmids was used to inoculate 5 mL of LB medium supplemented with the aforementioned antibiotics and incubated with agitation for 16 hours at 37°C. Subsequently, 2 mL of the overnight culture was used to inoculate 200 mL of LB medium with the selective antibiotics. When the culture reached OD_600_: 0.6, 100 µL of 1 M IPTG (isopropyl β-D-thiogalactoside) was added to half of the culture to induce the expression of the gene and the other half was used as control. All the assays were performed in triplicate and in darkness, in order to maximize carotenoid production.

### Vector construction for post-transcriptional gene silencing and over-expression of *DcLcyb1*

For post-transcripcional gene silencing, a 483 bp fragment of *DcLcyb1* cDNA (798 bp-1280 bp) was amplified from carrot leaf RNA with *LCYBF*S and *LCYBR*S (Table 1) and cloned in the *EcoR*I site of pUCpSS. Positive clones were analyzed by PCR and enzymatic digestion. After sequencing, clones with the *DcLcyb1* fragment in an antisense (AS) and in a sense (S) orientation with respect to the double 35SCaMV promoter (d35S) were digested with *Hind*III. The resulting d35S::*Lcyb*AS (antisense) and d35S::*Lcyb*S (sense) fragments were purified and cloned in the *Hind*III restriction site of the binary vector pBIN19, forming pB*Lcyb*AS and pB*Lcyb*S. For *DcLcyb1* over-expression, the complete *DcLcyb*1 coding sequence (DQ192190) cloned into pCR®8/GW/TOPO (Invitrogen) as described before (pCR8/*Lcyb1*), was recombined into the binary vector pMDC32 [46], following the manufacturer's instructions to produce pMDC32/*Lcyb1* in which *DcLcyb1* is inserted under the control of a d35SCaMV promoter.

### Agrobacterium tumefaciens-mediated transformation of *Daucus carota*

The binary vectors pB*Lcyb*AS, pB*Lcyb*S and pMDC32/*Lcyb1* were transformed into *Agrobacterium tumefaciens* (strain GV3101). *D. carota* transformation was achieved following the protocol described by Chen and Punja, 2002 [47]. Briefly, hypocotyl segments of 14 day-old seedlings were co-cultivated with *Agrobacterium* carrying the vector of interest, and placed on solidified MS media (4.4 g/L MS salts, 20 g/L sucrose and 0.7% agar) in darkness. After 2 days, the explants were transferred to solid MS medium containing 1 mg/L 2.4D for somatic embryogenesis induction and supplemented with 50 mg/L kanamycin (for pB*Lcyb*AS and pB*Lcyb*S) or 50 mg/L hygromycin (for pMDC32/*Lcyb1*) and 200 mg/L carbenicilin. After four weeks in darkness, the explants were placed on solidified MS medium containing 0.5 mg/L 2.4D, 100 mg/L kanamycin (for pB*Lcyb*AS and pB*Lcyb*S) or 100 mg/L hygromycin (for pMDC32/*LCYB1*) and 200 mg/L carbenicilin in photoperiod conditions (16 hrs light, 115umol/m2/sec). Antibiotic-resistant embryos were transferred to MS media in the absence of hormones to induce the development of shoots. After six months, transformed plantlets were transferred to soil in a temperature and photoperiod controlled greenhouse (16 hrs light, 115 umol/m2/sec). PCR amplification of *hptII* was carried out to select transgenic lines. *DcLcyb1*qRT was performed using *Lcyb1*Fq and *Lcyb1*Rq primers to select over expresser transgenic plants. Lines with reduced levels in *DcLcyb1* expression were obtained through conventional RT-PCR using previously described *hptII* primers and *FLcybi*: (5′-gggttagcggtagcacaac) and *RLcybi* ( 5′-gcctctgcctgtactccctc) primers, which amplify a 760 bp fragment that contains the 483 bp fragment used as target to induce silencing of the gene.

### DcLCYB1 sub-cellular localization

The full length *DcLcyb1* coding sequence was amplified without the stop codon from a sequenced pCR8/*Lcyb1* clone using Elongase enzyme and *Lcyb*F and *LCYB*R primers (Table 1) and subcloned into PCR8 (Invitrogen). Positive clones were sequenced in Macrogen Corp. Gateway technology was used to recombine pCR8/*Lcyb1st* into the binary vector pMDC85 [46] to obtain the chimeric protein LCYB1:GFP directed by ad35SCaMV promoter. The recA:YFP (kindly provided by Dr. Lee Meisel) harbors the chloroplastic destination peptide in frame with the YFP gene [48]. The constructs were transiently expressed in leaves of 2 month-old *Nicotiana tabacum* plants by agroinfiltration according to [49].The samples were visualized in an inverted Epifluorescence Microscope (IX-70, Olympus America Inc., Melville, NY). The GFP signal was measured at Ex 480/30X, Dichroic 505DCLP, Em 535/40mand processed with LSM5 Image Browser and Adobe Photoshop software.

### Carotenoid extraction and High Performance Liquid Cromatography (HPLC)

Carotenoids from wild-type and transgenic carrots were extracted from 100 mg of leaves or roots with 1 ml of hexane/acetone/ethanol (2∶1∶1 v/v) as described in [41]. Two successive extractions were performed to remove carotenoids until the tissue was blanched. The extract was dried with N_2_ and resuspended in 1 ml of acetone. Carotenoids from *E. coli* BL21 complemented cells were extracted from 70 mL of liquid cultures centrifuged at 5000 rpm for 15 minutes. The bacterial pellet was washed twice with cold sterile water and resuspended in 1 mL of sterile water with 500 µL of glass pearls. After vigorous mixing, 1 mL of acetone was added. Afterwards, the solution was again mixed vigorously and centrifuged at 4000 rpm for 5 minutes. The collected aqueous phase was washed twice with acetone and mixed vigorously with 4 volumes of petroleum ether. This solution was centrifuged and the upper phase collected in glass tubes, dried with gaseous nitrogen, resuspended in 100 µL of acetone and kept at −80°C until total carotenoid and carotenoid composition analysis was performed. Total carotenoids from plants and bacteria were measured by spectrophotometry at 474 nm in Shimadzu HPLC equipment (LC-10AT) with a diode array and the data analysis was carried out with the LCsolutions® software program. For phytoene measurements, chromatograms at 285 nm were obtained. These pigments were separated by a HPLC using a RP-18 Lichrocart125-4 reverse phase column (Merck®),utilizing a acetonitrile:methanol:isopropanol (85∶10:5 v/v) mix as a mobile phase with a 1 ml/min flow rate at room temperature in isocratic conditions. The elution spectra of each maximum were obtained using a diode array detector. The carotenoids were identified according to their absorption spectra, retention time and comparison with specific pigment standards, which was corroborated by comparison with the Carotenoids Handbook [50], [51]. All operations were carried out in triplicate, on ice and dark conditions to avoid photodegradation, isomerization and structural changes of carotenoids.

## Results

### *In silico* analysis and sub-cellular localization of DcLCYB1

**Figure 2:**
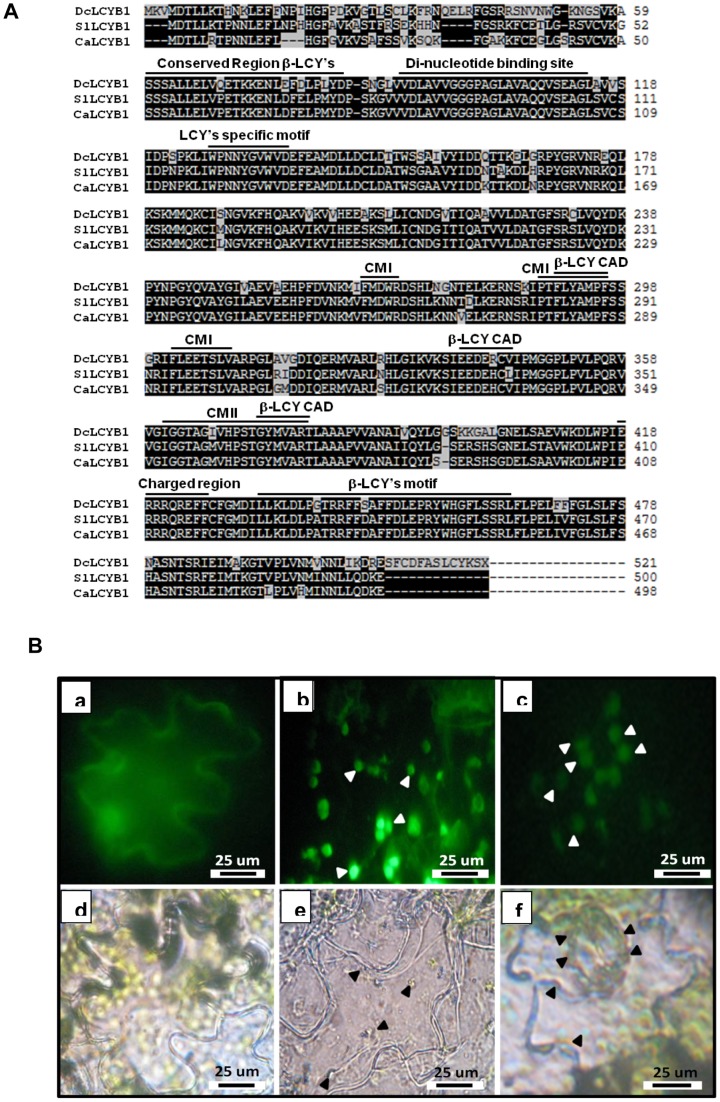
Comparative alignment and sub-cellular localization of DcLCYB1. (A) The alignment was created using ClustalW. Numbers on the right denote the number of amino acid residues. The amino acid residues which are identical in all sequences are shown in white text on a black background, whereas different residues are shown in black text on a white background. Characteristic regions of plant β-LCYs are indicated above the DcLCYB1 sequence: Conserved β-LCY region, Di-nucleotide binding site, Cyclase motifs (CM) I and II, Charged region and β-LCY motif. Domains described as essential for β-LCY activity are shown as β-LCY CAD (Catalytic Activity Domain). A plant LCY specific motif is also highlighted. SlLCYB1: *Solanum lycopersicum* lycopene β-cyclase 1; CaLCYB1: *Capsicum annuum* lycopene β-cyclase 1. (B) Subcellular localization of DcLCYB1. Leaves of two-month old tobacco plants were agroinfiltrated with *A. tumefaciens* carrying (a) pCAMBIA 35S::GFP, (b) pMDC85-*LCYB1* and (c) *recA*::YFP (positive control). After 4 days, epidermal peels were observed by epi-fluorescence microscope. (a) pCAMBIA 35S::GFP – a cytoplasmic localization of soluble GFP is visible. (b) pMDC85-*LCYB1* - the punctuate fluorescence is indicative of a chloroplastic localization of DcLCYB1-GFP. (c) pBI-*recA*- the punctuate fluorescence is indicative of a chloroplastic localization of *recA*::YFP. (d, e, f) Bright field images of a, b, c, respectively. All images were taken with 40x augmentation and fluorescence was observed after excitation at 489 nm.

The sequence analysis shown in Figure 2A indicates that DcLCYB1 has conserved motifs related to lycopene β-cyclases such as a cyclase motif I and II (CMI and CMII), a LCY specific motif, a conserved region β-LCY, a di-nucleotide binding site, a charged region and the β-LCY motif, β-LCY CAD region (Catalytic Activity Domain), domains described as essential for lycopene β-cyclase activity [17], [22], [23], [52].

DcLCYB1 has high amino acid identity with β-lycopene cyclase of tomato (SlLCYB1, 82.4%, [28] and pepper (CaLCYB1, 80.7%, [22]. The most variable region of DcLCYB1 is found at the N-terminus (Figure 2A) in which a plastid targeting sequence of 68 amino acids is predicted (ChloroPv1.1®).

To confirm these *in silico* findings, a DcLCYB1:GFP fusion protein was constructed under the control of a d35SCaMV promoter in order to determine the sub-cellular localization of the enzyme experimentally. The DcLCYB1:GFP fusion protein was transiently expressed in leaves of 2 month-old tobacco. Epifluorescence laser microscopy of leaves post-infiltration showed that DcLCYB1:GFP has a chloroplastic localization similar to that of the known chloroplast-targeted protein, RECA fused to YFP (Figure 2B; [48]).

### Expression analysis of *DcLcyb1* in carrot leaves and roots

**Figure 3:**
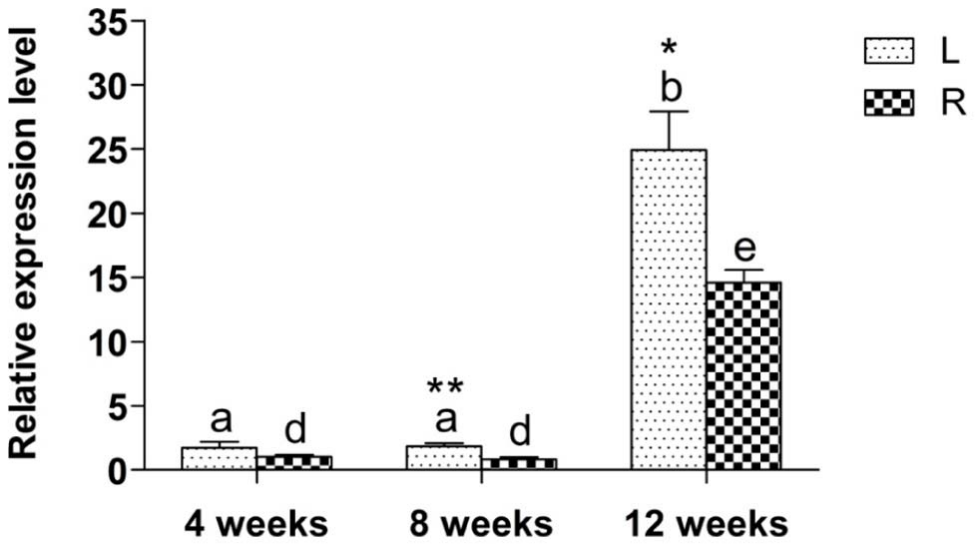
Expression levels of *DcLcyb1* in leaves and storage roots during *D. carota* development. Expression analysis was carried out in 4, 8 and 12 week-old plants. Different letters indicate significant differences between developmental stages and between leaves and roots. Asterisks indicate significant differences between both organs at the same stage of development. *Ubiquitin* was used as a normalizer, while data of leaves of 4 week-old plants were used as calibrator. A non-paired one and two tailed t-test (p<0.05) was performed.

A *DcLcyb1* gene was identified [42] and expression analysis in the carrot storage root has been performed previously [39], [41], [43]. The expression of *DcLcyb1* from the Nantaise cultivar increases throughout root development and is one of the genes that exert the most highly induction in leaves and in the storage root [41]. In addition, a positive correlation between *DcLcyb1* expression and β-carotene accumulation was observed during storage root development [41]. Here, we directly compared the expression of *DcLcyb1* in leaves and storage root in different developmental stages of carrot. As shown in Figure 3, the gene was more highly expressed in mature plants, reaching 25 fold and 14 fold higher levels in leaves and storage roots, respectively, compared to young plants. The expression of *DcLcyb1* was 1.6 fold greater in leaves compared to roots in a mature stage.

### Functional analysis of *DcLcyb1* by means of heterologous complementation

**Figure 4:**
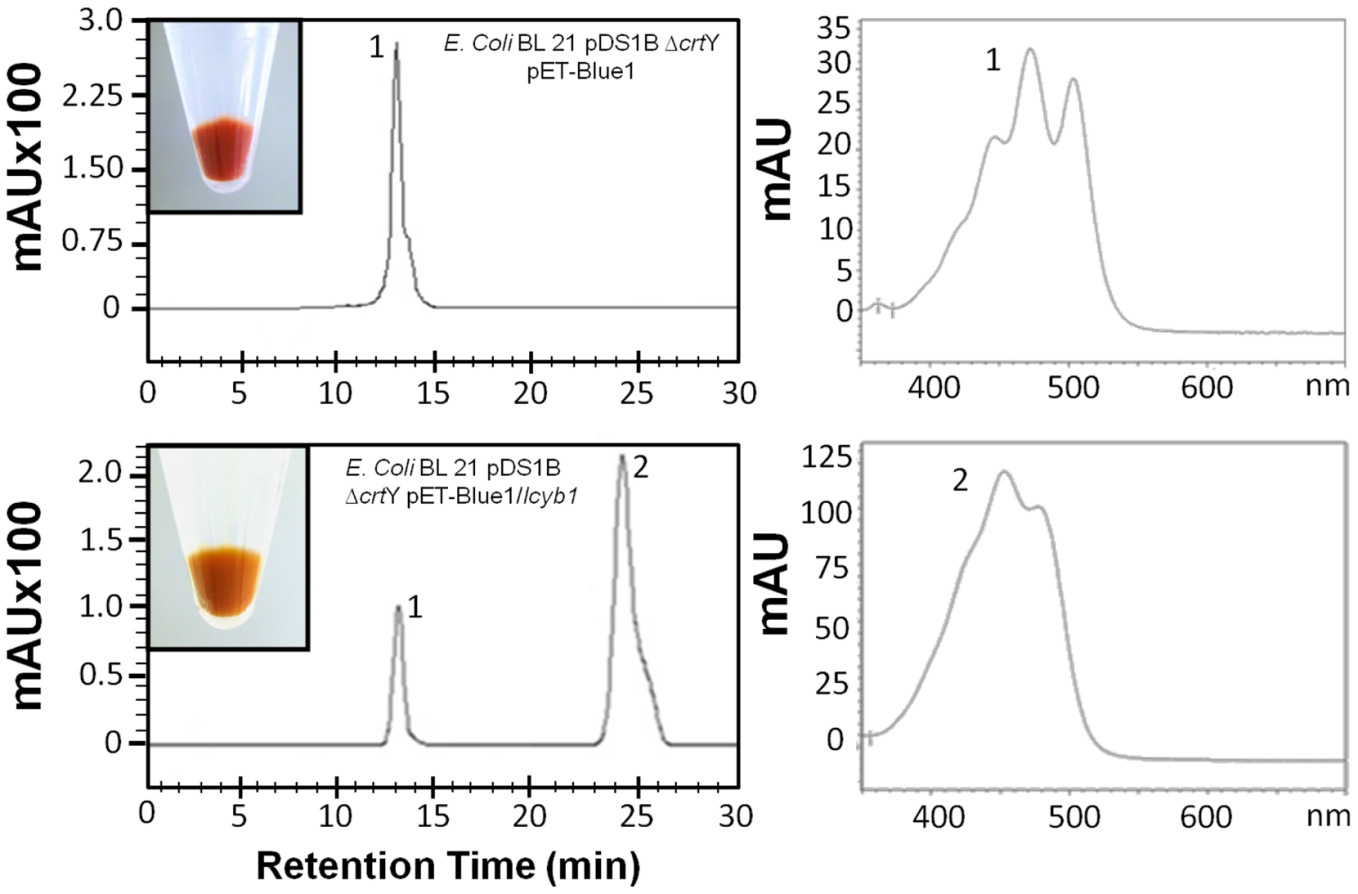
Reverse phase HPLC analysis of carotenoids accumulated in *E. coli BL21* strain complemented with *DcLcyb1*. Carotenoids were extracted from liquid bacterial BL21 cultures harboring pDS1BΔcrtY and transformed with either pET-Blue1 (upper pannel) or pET-Blue1/*Lcyb1* (lower pannel). The bacterial pellet from each transformed strains after complementation are shown in boxes in each chromatogram. Chromatograms show that both lycopene and β-carotene are present in the strain transformed with *DcLcyb1*, while the control was not able to restore the mutation of *crtY* gene in the strain, producing only lycopene. The spectra of lycopene and β-carotene are shown in the right-hand side of the figure with numbers 1 and 2, respectively. Peak 1 corresponds to lycopene, which presents a retention time of 13 minutes and peak 2 corresponds to β-carotene with a retention time of 24 minutes [72].

In order to evaluate the function of *DcLcyb1*, an *in vivo* analysis through heterologous complementation of the pDS1B Δ*crtY E. coli* BL21 gold strain [45] was performed. This mutant strain carries a plasmid (pDS1B) harboring the genes from *Erwinia uredovora* necessary for β-carotene synthesis, and a mutation in *Lcyb* (*crtY*) gene leads to the accumulation of lycopene. The strain was co-transformed with pET-Blue1/*DcLcyb1* and as a negative control with pET-Blue1. Carotenoids were extracted from liquid bacterial cultures (48 hours culture), and analyzed by reverse phase HPLC. The chromatogram obtained from mutant strains co-transformed with pET-Blue1/*Lcyb1* in the presence of IPTG showed a peak with a characteristic absorbance spectrum corresponding to β-carotene that is absent in the control (Figure 4). The quantification of the HPLC chromatogram showed that the complemented strain (pDS1BΔ*crtY*/pET-Blue1/*DcLcyb1*) presents significantly higher levels of β-carotene accompanied by a diminished level of lycopene compared to the control strain pDS1BΔ*crtY*/pET-Blue1 (not shown). Our findings show that *DcLcyb*1 is able to restore the carotenoid biosynthetic pathway in the pDS1B Δ*crtY E. coli* strain, leading to β-carotene production.

### Over-expression of *DcLcyb1* in *D.carota* increases carotenoid levels and affects endogenous carotenogenic gene expression

**Figure 5:**
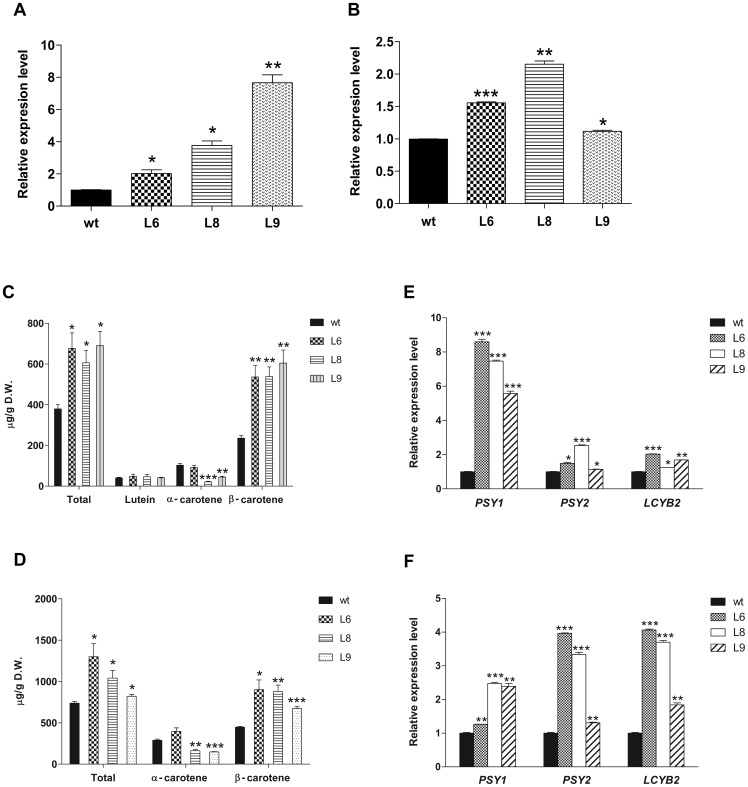
Over expression of *DcLcyb1* increases carotenoid and β-carotene levels and expression of key carotenogenic genes. Relative expression of *DcLcyb1* gene in L6, L8 and L9 from (A) leaves and (B) storage roots of transgenic carrot plants. *Ubiquitin* was used as normalizer in qRT measurements. Carotenoids of *D. carota* L6, L8 and L9 transgenic lines were extracted from (C) leaves and (D) storage roots and carotenoid composition was determined by spectrophotometry and High Performance Liquid Chromatography. Relative expression of *DcPsy1*, *DcPsy2* and *DcLcyb2* in transgenic carrot lines from (E) leaves and (F) storage roots. *Ubiquitin* was used as normalizer in qRT measurements. For gene expression and carotenoid analysis three months-old plants were used. Columns and bars represent the means and SE (n = 3). Asterisks indicate significant differences between transgenic lines and the wild type plant. Non-paired one and two tailed t-tests (p<0.05) were performed for all the transgenic lines and the wild type plant.

To evaluate the function of *DcLcyb1 in planta*, the ORF was cloned in the binary vector pMDC32 and transferred by Agrobacterium-mediated transformation into *D. carota*. The transformed lines were verified by PCR amplifying *hptII* gene, and three independent transgenic lines were selected and subjected to qRT, to evaluate *DcLcyb1* expression (Figure 5A and B). In leaves, the expression of *DcLcyb1* was 2 to 8 fold greater than that observed in wild type plants (Figure 5A), and in roots of the transgenic lines, an increment of 1.1 to 2.3 fold in the expression of *DcLcyb1* related to the roots of wild type plants was obtained (Figure 5B). On analyzing carotenoid pigments, the over expression of this gene induced increments of 1.6 to 1.8 fold in total carotenoids and 2.6 to 2.8 fold in β-carotene levels in leaves, compared to wild-type plants (Figure 5C). A modification of the other branch of carotenoid biosynthesis was observed in leaves of some lines, where α-carotene levels were reduced until -5 fold (Figure 5C), reflecting a possible function of *DcLcyb1* in both branches of the carotenogenic pathway, redirecting the flux to the synthesis to β-carotene. In the storage root, the total carotenoid and β-carotene levels in over expresser lines increased 1.1 to 1.8 fold, and 1.2 to 2 fold, respectively, with respect to the wild-type plants, while α-carotene levels changed between -0.5 to 1.5 fold (Figure 5C and D).

**Figure 6:**
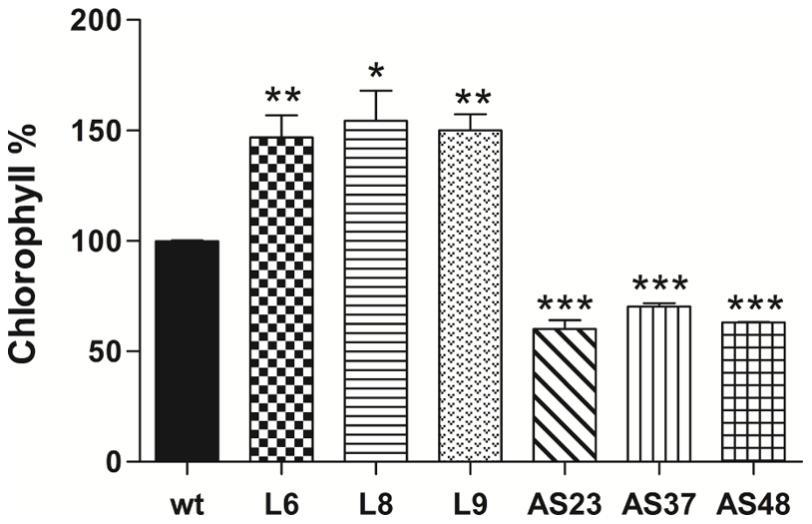
Chlorophyll content in over expresser and silenced *DcLcyb1* lines of carrot plants. Chlorophyll amount in leaves of over expresser and silenced lines was determined by spectrophotometry and HPLC. The chlorophyll amount in wild-type was set at 100%. For this analysis, leaves of three month-old plants were used. Columns and bars represent the means and SE (n = 3). Asterisks indicate significant differences between transgenic lines and the wild type plant. Non-paired one and two tailed t-tests (p<0.05) were performed for all the transgenic lines and the wild type plant.

As expected, chlorophyll content was also increased in the transgenic lines (Figure 6) because chlorophyll and carotenoid synthesis is coordinately regulated [53], [54]. Phytoene levels did not change in transgenic lines related to wild type plants (not shown), despite the increment in expression levels of *DcPsy1* and *DcPsy2* (Figure 5E and F). This could be associated with the increment in *DcLcyb1* and *DcLcyb*2 expression that may enhance the flux to β-carotene synthesis. The phenotype of whole and cross-sectioned transgenic storage roots, shown in Figure S1, showed that they differed in thickness and color with respect to the wild-type carrots, and correlated with the carotenoid content, especially in L6 (Figure 5D). To analyze the effect of *DcLcyb1* over-expression in the carotenogenic pathway, the transcript levels of endogenous carotenogenic genes were evaluated. *dcPsy1*, *Dcpsy2* and *DcLcyb2* genes were significantly induced in leaves and storage roots of the three transgenic lines (Figure 5E and F). In leaves, induction of *Dcpsy1* was greater than that of the paralog gene, *Dcpsy2* (5.8–8.4 fold versus 1.2–2.5 fold, respectively). In roots, L6 and L8 transgenic lines, which presented the greatest increase in carotenoid levels in carrot roots, the *Dcpsy2* and *DcLcyb2* genes were induced 3.5 to 4 fold related to wild-type storage roots, whereas *Dcpsy1* was induced by only 1.2 to 2.5 fold. These results show that the over expression of *DcLcyb1* in carrot produces metabolic changes and transcriptional modulation of the carotenogenic pathway, which in turn leads to an increment in total carotenoids and β-carotene.

### Post-transcriptional gene silencing of *DcLcyb1* affects carotenoid levels and endogenous carotenoid gene expression

**Figure 7:**
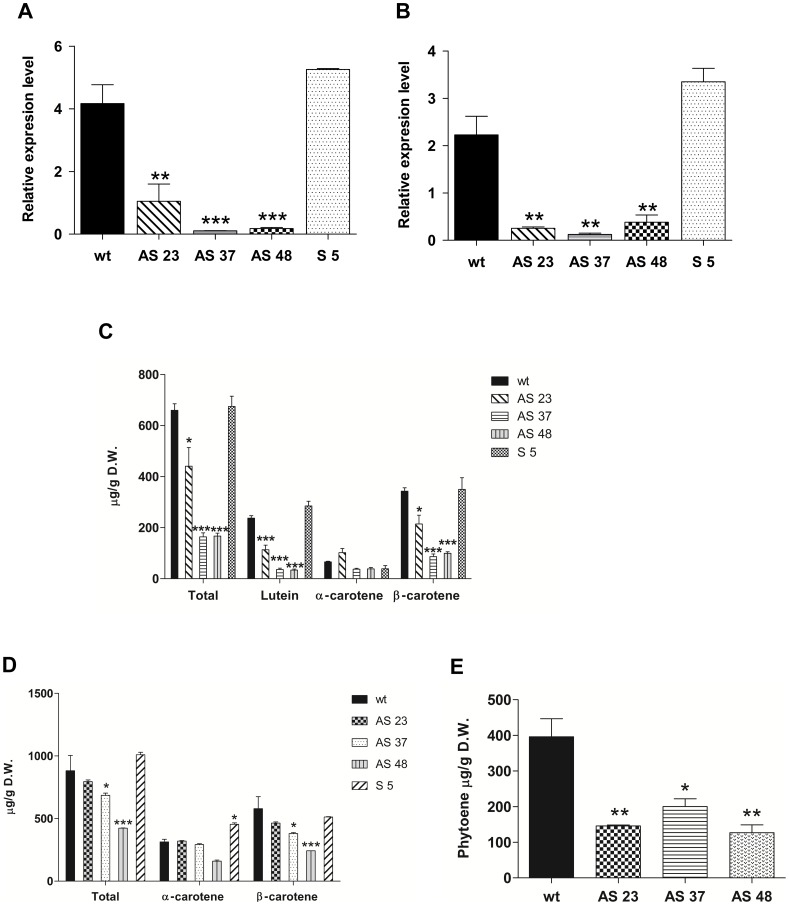
*DcLcyb1* silenced lines have diminished carotenoid and β-carotene levels in leaves and roots. *DcLcyb1* transcript levels in (A) leaves and (B) storage roots in carrot transgenic lines were evaluated by qRT. *Ubiquitin* was used as normalizer. Carotenoid quantification in (C) leaves and in (D) storage roots of wild-type (WT) and silenced lines was determined by spectrophotometry and HPLC at 474 nm. (E) Phytoene in storage roots of wild-type (WT) and silenced lines was quantified by HPLC at 285 nm. For gene expression and carotenoid analysis, three months-old plants were used. Columns and bars represent the means and SE (n = 3). Asterisks indicate significant differences between transgenic lines and the wild type plant. Non-paired one and two tailed t-test (p<0.05) were performed for all the transgenic lines and the wild type plant.

A post transcriptional gene silencing strategy was used to confirm the previous results, and to evaluate whether this gene has an organ specific function in carrot. Six silenced lines were obtained through qRT and three of them were analyzed in detail. The AS23, AS37 and AS48 silenced lines showed between 80% and 95% reduced expression of *DcLcyb1* in leaves and roots (Figure 7A and B). In lines with reduced expression of *DcLcyb1*, the carotenoid levels were reduced by 70% in leaves and by 55% in the storage root, and β-carotene levels were reduced by 77% and 58% in leaves and roots, respectively (Figure 7C and E). In addition to carotenoid reduction, a pronounced reduction in storage root thickness and color was obtained in transgenic silenced lines respect to wild type carrots (Figure S1) in which the narrow root reaches half of wild type thickness. As changes in root phenotype and in carotenoid levels in leaves and storage roots of the silencing lines were observed, these results suggest that *DcLcyb1* is involved in carotenoid biosynthesis in the entire carrot plant.

**Figure 8:**
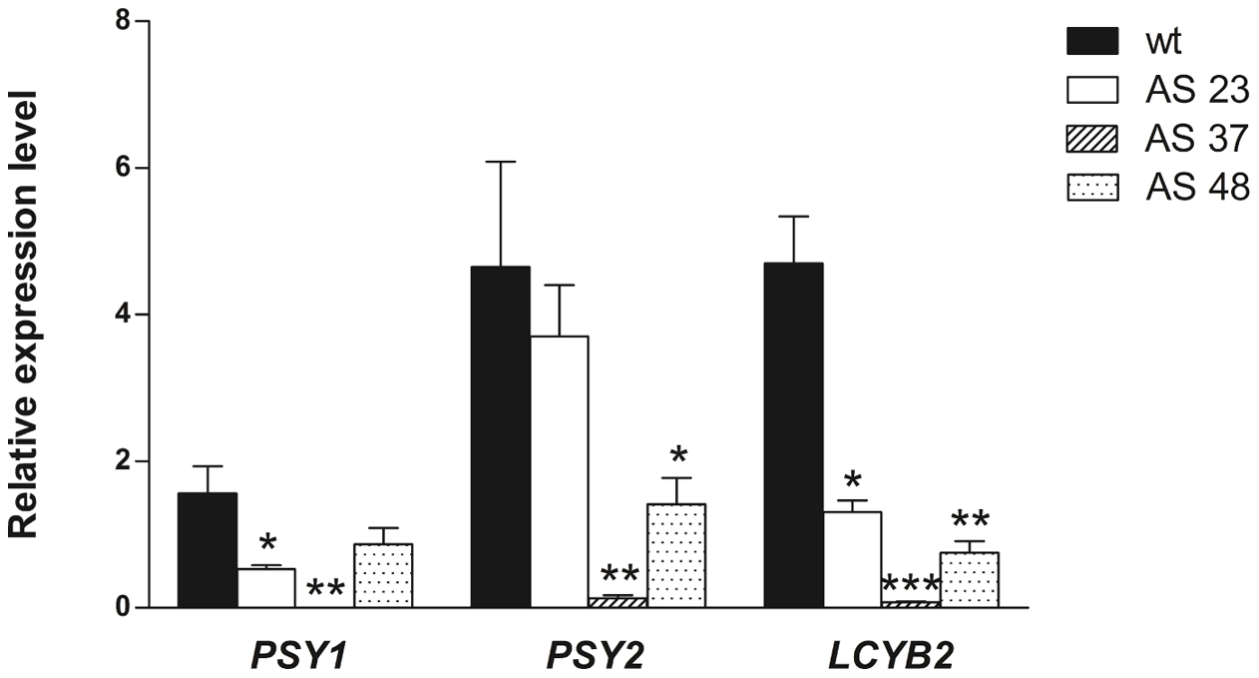
Expression of *DcPsy1*, *DcPsy2* and *DcLcyb2* decreases in transgenic carrots with reduced *DcLcyb1* expression levels. Relative expression of *DcPsy1*, *DcPsy2* and *DcLcyb2* in storage roots from six months-old AS23, AS37 and AS48 transgenic lines with reduced levels of *DcLcyb1* expression were evaluated by qRT. *Ubiquitin* was used as normalizer. Columns and bars represent the means and SE (n = 3). Asterisks indicate significant differences between transgenic lines and the wild type plant. Non-paired one and two tailed t-test (p<0.05) were performed for all the transgenic lines and the wild type plant.

The carotenoids from the other branch, especially lutein, were also reduced in leaves of the silenced plants (Figure 7C). These results suggest that the endogenous carrot *DcLcyb1* is required for the correct synthesis of carotenoids of both branches (β-carotene and α-carotene) in leaves. However, in roots, the reduction in *DcLcyb1* expression did not significantly affect the amount of α-carotene in silenced lines (Figure 7D), but the phytoene level was reduced significantly (Figure 7E) in a direct correlation with the endogenous gene expression of *DcPsy1* and *DcPsy2* (Figure 8). A 30% to 40% decrease in chlorophyll levels was obtained in leaves of carrots with reduced *DcLcyb1* levels (Figure 6), which correlates with the results obtained for the over expresser *DcLcyb1* lines. In these analyses, the S5 line transformed with the sense construct (pB*Lcyb*S) was used as silencing control and exhibited a very similar expression level of *DcLcyb1* compared to the wild-type, and consequently, the production of total carotenoids and β-carotene were not significantly different.

The effect of *DcLcyb1* silencing on the expression of other genes involved in the carotenogenic pathway was analyzed in the storage roots of transgenic lines (Figure 8). In storage roots, the transgenic lines presented a general decrease in carotenogenic transcript levels, especially in *DcPsy2* and *DcLcyb2* in AS37 and AS48 where a reduction of 98% (48 fold) to 69% (3.2 fold) was observed for *DcPsy2* and between 98% (48 fold) and 83% (6 fold) for *DcLcyb2.* This result correlated with the lower carotenoid and phytoene accumulation in these silenced carrot roots (Figure 7D and E).

Therefore, the reduction of *DcLcyb1* expression in carrot led to consistent effects over the expression of key carotenogenic genes which could be in part responsible for the alterations in the carotenoid accumulation observed in those lines.

## Discussion

The enzymatic reaction that gives rise to α-and β-carotene pigments is a key regulatory branching point performed by the lycopene β-and ε-cyclases (LCYB and LCYE) in plant plastids [10], [20], [38], [55], [56]. LCYB is encoded by single genes in several plants, including Arabidopsis, maize and rice [17], [24], [26], [57], or by small gene families in others, like tomato, pepper, papaya and orange [16], [28], [31], [33], [42], [52], [58].In most of these plants, organ specific functions of *LCYB* genes have been described.

In carrot, β-carotene and α-carotene are the major carotenoid components in a mature storage root, whose levels increase significantly during root development reaching 600 µg/g dry weight (dw) and 300 µg/g dw, respectively [43], [59]. This increase indicates an important stimulation of the β,β-cyclization of lycopene in the carrot carotenogenic pathway during storage root development. In carrot, two *Lcyb* genes have been identified, named *DcLcyb1* and *DcLcyb2.* Quantitative analysis indicated that *Lcyb1* expression is induced in storage roots during carrot development whilst *Lcyb2* is not [41]. The expression of *DcLcyb1* correlates with the accumulation of carotenoids in mature carrot roots [41]. *DcLcyb1* is also induced in leaves during carrot development, and reaches a higher level of induction in these organs than in the storage root in a mature plant (Figure 3), although the amount of carotenoids in leaves remains constant [41]. This could be associated with the photo-protective function in which carotenoids are involved in leaves. Carotenoids protect cells from the effects of excessive light exposure, which rises the carotenoid turn over in leaves which in turn could affect the expression level of the corresponding genes.

In roots, *DcLcyb1* expression increases 14-fold during development, and is one of the most highly-induced carotenogenic genes (Figure 3; [41]) suggesting that *DcLcyb1* is an important control point in this pathway. This gene expression profile correlates with that in the orange stage of Satsuma mandarin and Valencia orange fruit ripening, where a simultaneous increase in the expression of *CitPsy*, *CitPds*, *CitZds*, *CitLcyb*, *CitHyb*, and *CitZep* genes led to a high accumulation of xanthophylls in the flavedos and juice sacs of the citrus fruits [60].

Here, we functionally characterized the lycopene β-cyclase 1 gene of *D.carota* in order to determine whether this gene is involved in the biosynthesis of carotenoids in carrot. First, we analyzed whether DcLCYB1 possesses the conserved regions characteristic of plant LCYBs, by comparing the amino acidic sequence of DcLCYB1 with the chloroplast specific SlLCYB (*Solanum lycopersicum* LCYB) and CaLCYB (*Capsicum annuum* LCYB) which belong to plants that present high carotenoid accumulation. The *in silico* analysis of DcLCYB1 showed that it harbors the conserved motifs of the other plant LCYBs (Figure 2A). For example, the three β-LCY CAD regions that were reported as essential for lycopene β-cyclase catalytic activity are fully conserved in DcLCYB1 [14], [22], [23]. In the “conserved region β-LCYs”, which has been proposed as essential for the association of LYCB to membrane components and also for its catalytic activity [14], [22], there are four amino acid changes in carrot LCYB1 compared to the sequences of SlLCYB and CaLCYB. Specifically, these are a glutamic acid instead of a proline at position 60, a glutamine instead of an asparagine at position 68, an asparagine instead of a glutamine at position 70 and a leucine instead of metionine at position 73 (Figure 2A). In addition, the “β-LCYs motif” also possesses a single amino acid change that affects the amino acid charge at position 446, where an aspartate conserved in pepper and tomato sequences is replaced by a serine in the DcLCYB1 sequence from carrot (Figure 2A). However, the possible amino acidic changes identified *in silico*, did not alter the functionality of DcLCYB1, as shown by *in vivo* experiments carried out in the present article. The specific impact of the different amino acid substitutions identified *in silico* on lycopene β-cyclase activity requires further characterization using site directed mutagenesis.

Bioinformatic analysis using ChloroPv1.1® showed that the DcLCYB1 protein sequence presents the signal peptide in its N-terminal, as found in other plant carotenogenic enzymes [8], [19], [21]. In agreement with the *in silico* analysis, the sub-cellular localization carried out by means of a chimeric LCYB1-GFP fusion protein that was transiently expressed in tobacco leaves, confirmed the chloroplast localization of this enzyme (Figure 2B). Carotenoid enzymes are found in plastids (especially in chloroplasts and chromoplasts) of plant cells [38], where they exert their function. This result supports the proper organelle localization for this enzyme and suggests that DcLCYB1 functions correctly, which was directly proven by means of the heterologous complementation system in *E. coli*. This methodology has been reported previously to be useful in determining carotenogenic protein functionality [31], [52], [61]. As expected, the DcLCYB1 protein was able to restore the normal course of the carotenogenic pathway leading to the production of β-carotene using lycopene as substrate in *E. coli* (Figure 4). In addition to validating the utility of this heterologous system for determining the activity of a plant carotenogenic enzyme, these results proved that the *DcLcyb1* encodes for an enzyme with lycopene β-cyclase activity.

In order to determine the functionality of this gene in plants, we used two different approaches, over-expression and post-transcriptional gene silencing of *DcLcyb1*in carrot, since these techniques have been used elsewhere to evaluate gene function *in planta* [28], [33], [40], [62]. The stable over-expression of *DcLcyb1* in carrot led to an increase in total carotenoids and β-carotene levels in leaves and carrot roots, respectively (Figure 5C and D), which is correlated with *DcLcyb1* transcript levels in leaves and roots (Figure 5A and B). The proportion of β-carotene and α-carotene is mostly determined by the comparative amounts and/or activities of the LCYB and LCYE enzymes [26], [28], [63], [64], [65]. Transgenic carrots that overexpress *DcLcyb1*, redirect the carotenoid flux to β,β-carotene, diminishing the amount of β,ε-carotene molecules.

In wild type and transgenic carrots, the carotenoid levels in roots are higher than in leaves although the expression of *DcLcyb1* is lower in this organ than in leaves (Figures 3, 5A and B). This was also observed for other carotenogenic genes [41] because total carotenoid levels, including β-carotene accumulation, are determined by the total flux in the pathway in which enzymatic activity and stability may also exert an important role. In addition, in leaves, the carotenoid levels are the net result of the synthesis and degradation of carotenoids exerted by light through photo-oxidative processes [66], whereas in roots the carotenoids are stored in stable structures (plastoglobuli and crystals), present in chromoplasts that could diminish the carotenoid degradation rate [40].

In addition, when *DcLcyb1* was over-expressed in carrot, a side effect on the transcript levels of endogenous carotenogenic genes was observed (Figure 5E and F). In carrot leaves and roots, the *DcPsy1*, *DcPsy2* and *DcLcyb2* genes were also induced in transgenic lines. Römer et al., 2000 [67] also obtained an induction in endogenous *SlPds*, *SlZds* and *SlLcyb* expression on overexpression of 35S::crtI in tomato. In contrast, the over expression of *AtLcyb* in tomato under a *Pds* promoter, did not affect significantly the expression of endogenous carotenogenic genes, although the fruits contained more lycopene and β-carotene[68]. Thus, a direct correlation between the increase of *DcLcyb1* transcript levels (Figure 5A and B), the total carotenoid and β-carotene levels (Figure 5C and D), and the up regulation in the expression levels of *DcPsy1*, *DcPsy2* and *DcLcyb2* (Figure 5E and F) is observed.

When a bacterial crtB gene under a root specific promoter was expressed in the white QAL carrot variety, an increment in crystal accumulated-carotenoids was obtained [40]. However, these carrot lines were enriched in carotenoid intermediates such as phytoene, phytofluene and ζ-carotene [40]. In *DcLcyb1* over expresser lines, phytoene was not altered, but the levels of the direct products of *Lcyb*, which include α-carotene and β-carotene, were significantly increased. This result suggests that *DcLcyb1* might act as a carotenogenic pathway regulator, possibly through an indirect positive feedback mechanism over the expression of other carotenogenic gene(s), which further increases carotenoid accumulation in transgenic carrot plants.

In over-expressing lines L6 and L8, which displayed the highest carotenoid and β-carotene level, the expression of *DcPsy2*, and not *DcPsy1*, was significantly induced in the storage root, which could also assist in raising the carotenoid level in this organ. Moreover, *DcPsy1* is induced in leaves of these transgenic lines, which suggests that the requirement of both organs regarding *DcPsy* induction through *DcLcyb1* should be different. Recently, we determined that *DcPsy2* is preferentially expressed during carrot root development, while *DcPsy1* is most highly expressed in mature leaves [41], findings which correlate with the results presented here.

On the other hand, post-transcriptional gene silencing of *DcLcyb1* showed that this gene is necessary for β-carotene synthesis in leaves and in roots of *D. carota* (Figure 7C and D), and especially in leaves, correlating with the highest endogenous expression level (Figure 3). The two transgenic lines (AS37, AS48), which presented the most substantial decrease in *DcLcyb1* expression levels in leaves and roots (more than 90% of gene silencing), showed up to a 77% reduction in β-carotene in photosynthetic organs and up to a 58% decrease in the storage root (Figure 7 C and D). In addition, we observed a significant reduction in lutein levels in leaves, although no differences were observed in the level of α-carotene in these organs (Figure 7C). This could be explained because the activity of other enzymes, such as DcLCYE and/or DcCHBX, might not be being affected by the reduction of *DcLcyb1* expression. Interestingly, phytoene levels decreased between 2 and 3 fold in roots of the transgenic lines with reduced levels in *DcLcyb1*, consistent with a reduction in the expression of *DcPsy1* and *DcPsy2*.

As mentioned above, carotenoids carry out a fundamental role during photosynthesis, being located in the photosynthetic membrane in carotenoid-chlorophyll complexes [10]. In addition, chlorophylls and carotenoids, that share GGPP as a common precursor, are coordinately regulated during de-etiolation and by the redox status [69], [70]. Light is a stimulus that induces the expression of some carotenogenic genes and simultaneously activates the expression of *Ppox* (protoporphyrinogen IX oxidase), a gene that encodes a light- harvesting chlorophyll a/b binding protein [54], [69]. The phytochrome system is involved in the control of carotenogenic gene expression, as a greater induction of *Psy* and *Lcyb* transcripts was detected after exposure to red-light than to blue-light [69]. In addition, changes in carotenoid composition or in enzymes itself, alters plastid development [71]. Our results indicate that an increment or reduction in carotenoids, affects directly and significantly the amount of chlorophyll in transgenic plants, suggesting that an increase or reduction in *DcLcyb1* expression, may alter the amount of a common precursor of both pathways. With these results, we suggest that *DcLcyb1* is required for carotenoid accumulation in the whole carrot plant, and that it does not have an organ-specific function in this specie, unlike that reported previously for tomato *SlLcyb* and *SlCycb* [28], [58], [64].

Moreover, the silencing of *DcLcyb1* caused a significant decrease in the expression of endogenous carotenogenic genes in carrot roots, particularly in *DcPsy2* and *DcLcyb2;* correlating with the level of reduction in *DcLcyb1* expression in the transformed carrot lines (Figure 8). The 483bp fragment used to induce *DcLcyb1* silencing has just 58% nucleotide identity with *DcLcyb2*. Therefore, the RNAi strategy employed in this work would not have an effect on the expression of *DcLcyb2* directly, and the reduction of *DcLcyb2* expression determined by qRT was a consequence of *DcLcyb1* silencing.

In conjunction with the alterations observed in the transgenic carrot plants which over-expressed *DcLcyb1*, our findings lead us to conclude that an increase or decrease in *DcLcyb1* transcript levels produces not only changes in carotenoid accumulation but also in the thickness of storage root and expression of key endogenous carotenogenic genes. Taken together, these results demonstrate that *DcLcyb1* encodes for a LCYB enzyme that is functional and a key player in the carotenogenic pathway in *Daucus carota*. Further studies could be focused on the identification and characterization of the *DcLcyb1* promoter to study the regulation of this gene during carrot development, in leaves and in the storage root.

## Supporting Information

Figure S1 **Storage root phenotype of** ***DcLcyb1*** **over expresser and silenced lines.** Pictures were taken in 3 months old representative carrots that were cultivated in a mix of soil and vermiculite (2∶1) in a growth chamber with cool-white fluorescent light (115 μmol m^−2^ s^−1^). Horizontal bar: 2 cm. Vertical bar: 3 cm.
(TIF)
