# Supplementary figures and images for: Levels of Lycopene β-Cyclase 1 Modulate Carotenoid Gene Expression and Accumulation in Daucus carota
Source: PLoS One. 2013 Mar 29;8(3):e58144. doi: 10.1371/journal.pone.0058144 (PMC3612080; doi:10.1371/journal.pone.0058144)

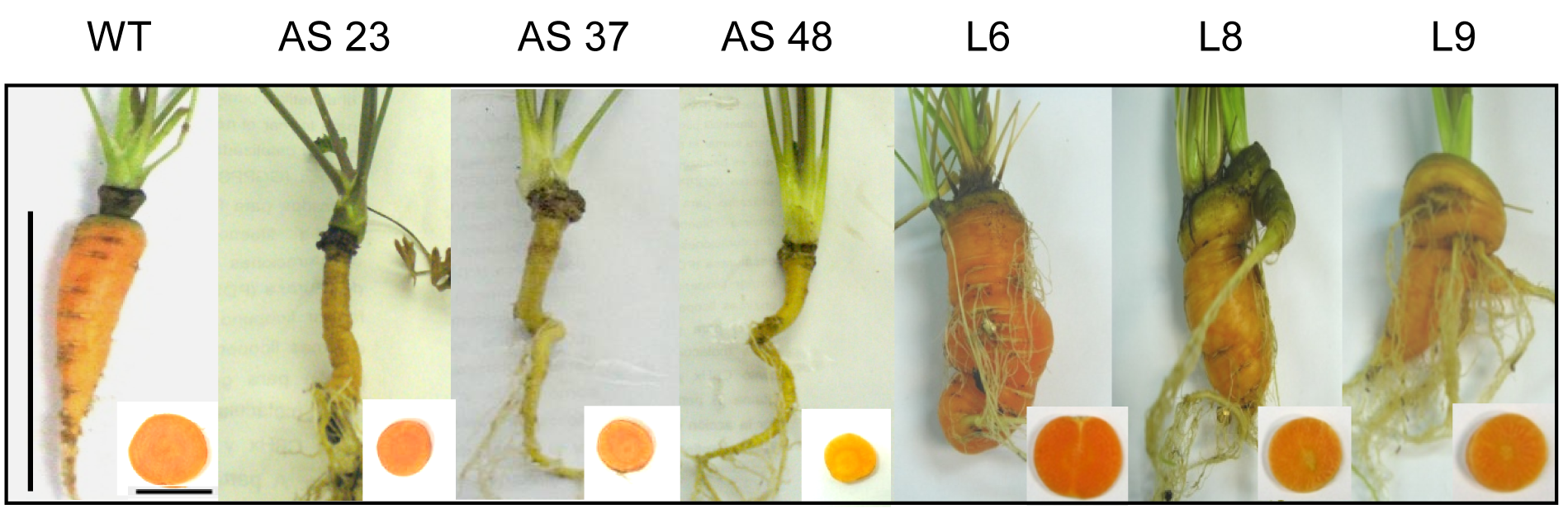

Supplement: Figure S1 — Storage root phenotype of DcLcyb1 over expresser and silenced lines. Pictures were taken in 3 months old representative carrots that were cultivated in a mix of soil and vermiculite (2∶1) in a growth chamber with cool-white fluorescent light (115 μmol m−2 s−1). Horizontal bar: 2 cm. Vertical bar: 3 cm. (TIF) [file pone.0058144.s001.tif]
